# Secondhand Smoke Exposure of Expectant Mothers in China: Factoring in the Role of Culture in Data Collection

**DOI:** 10.2196/24984

**Published:** 2021-10-07

**Authors:** Zhaohui Su, Dean McDonnell, Jaffar Abbas, Lili Shi, Yuyang Cai, Ling Yang

**Affiliations:** 1 Center on Smart and Connected Health Technologies, Mays Cancer Center School of Nursing University of Texas Health San Antonio San Antonio, TX United States; 2 Department of Humanities Institute of Technology Carlow Carlow Ireland; 3 School of Public Health Shanghai Jiao Tong University School of Medicine Shanghai China; 4 China Institute for Urban Governance Shanghai Jiao Tong University Shanghai China

**Keywords:** cancer, secondhand smoking, secondhand smoke, expectant mothers, pregnant women, China, transitional Chinese culture, “doing the month”, smoking, pregnancy, women, China, culture, behavior

## Abstract

Cancer is the leading cause of death worldwide. Tobacco smoking, including secondhand smoking, causes cancer and is responsible for over 22% of global cancer deaths. The adverse impacts of secondhand smoke are more pronounced for expectant mothers, and can deteriorate both mothers’ and infants’ health and well-being. Research suggests that secondhand smoke significantly increases expectant mothers’ risk of miscarriage, cancer, and other chronic disease conditions, and exposes their unborn babies to an increased likelihood of having life-long poor health. In China, a pregnant woman’s family members, such as her husband, parents, or in-laws, are the most likely people to be smoking around her. Due to traditional Chinese cultural practices, even though some expectant mothers understand the harm of secondhand smoke, they may be reluctant to report their family members’ smoking behaviors. Resulting in severe underreporting, this compromises health experts’ ability to understand the severity of the issue. This paper proposes a novel approach to measure secondhand smoke exposure of pregnant women in the Chinese context. The proposed system could act as a stepping stone that inspires creative methods to help researchers more accurately measure secondhand smoking rates of expectant mothers in China. This, in turn, could help health experts better establish cancer control measures for expectant mothers and decrease their cancer risk.

## Background

Cancer is the leading cause of death worldwide [1]. Tobacco smoking, including secondhand smoking, causes cancer and is responsible for over 22% of global cancer deaths [2]. In 2017 alone, 62.9 million disability-adjusted life years were lost in China due to cancer [3]. With the current prevalence of smoking, the situation is expected to worsen in the future [4]. China has the largest population of tobacco smokers worldwide—one in every three smokers across the globe is Chinese [4]. Different from other human addictions (eg, opioids), tobacco smoking not only harms smokers’ health but also harms the health of individuals exposed to secondhand smoke [5].

While smoking has been declining in China (eg, among Chinese adults aged 30-69 years, 11.0% of smokers quit in 2010 compared to 4.2% quit rates in 1996), secondhand smoking remains a persistent public health issue that harms people’s health and well-being [6]. Secondhand smoking can be understood as nonsmokers’ exposure to smoke from tobacco products due to regular contact with smokers in close proximity to them [7]. Individuals subjected to secondhand smoke face unique health challenges despite not smoking cigarettes. They are exposed to the same set of detrimental health consequences associated with tobacco smoking, ranging from physical health consequences (eg, an elevated risk of cancer) to pronounced psychological health challenges [8].

## Danger of Secondhand Smoke for Expectant Mothers

**Figure 1:**
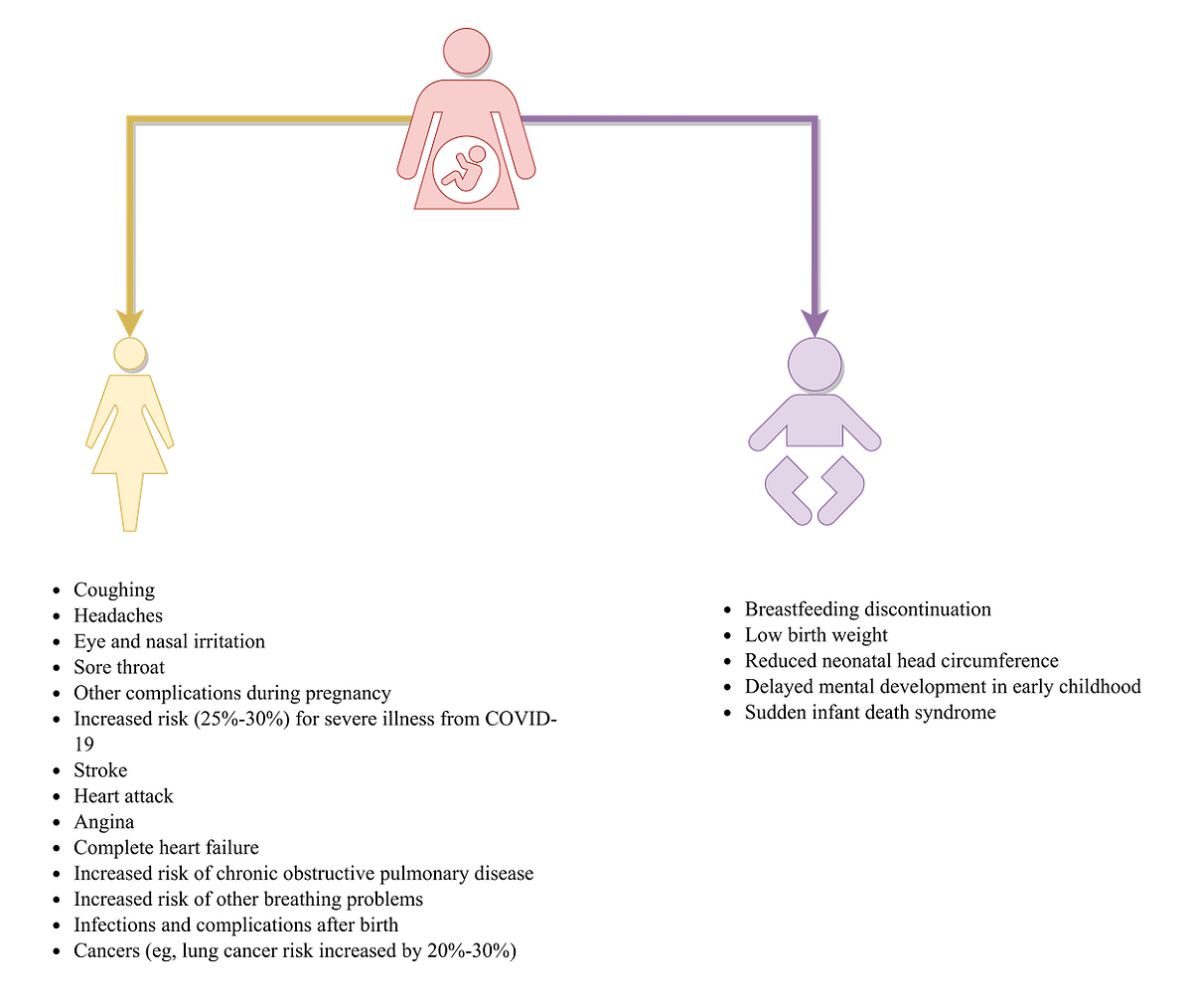
A schematic representation of the increased health risks of secondhand smoking for expectant mothers and their infants.

Recent evidence shows that Chinese women exposed to secondhand smoke often experience a significant decline in cognitive functions, such as memory, that can last up to two years [9]. However, the situation might be worse for expectant mothers. Women may experience various health issues during pregnancy including venous thromboembolism, diabetes, hypertension, and heart disease; in addition, women are at increased risk of domestic violence when pregnant [10-12]. Additionally, for this community, exposure to toxic materials often results in harm to both the women themselves and their unborn babies [13-15]. In other words, exposure to harmful substances, such as toxic secondhand smoke, harms pregnant women at a time when health risks are more likely to affect their long-term health outcomes, in addition to causing substantial harm to their unborn babies’ health [16-18]. Furthermore, secondhand smoke and its adverse effects are associated with an increased risk of infant mortality, including sudden infant death syndrome [19] (Figure 1).

## “Doing the Month”: A Unique Risk Factor for Secondhand Smoke Exposure

Secondhand smoking might have an even more significant impact on expectant Chinese women due to cultural practices. In China, it is common practice for partners, parents, or in-laws to take care of the pregnant woman during and beyond pregnancy to meet her basic needs [20]. Owing partially to traditional Chinese culture and social norms, immediately after women give birth to their babies, these helpers are also expected to attend to the needs and wants of women and their newborn infants during the “doing the month” ritual [21]. When followed stringently, “doing the month,” a traditional Chinese cultural practice that dates back more than 2000 years, requires women to follow an extensive list of rules. These rules include not leaving the house, refraining from contacting water or wind (eg, not washing one’s hair, taking full-body showers, or opening the window), and not consuming foods that have a “cold” nature, among other things, for a full month [22]. In a recent study of 2615 Chinese women, researchers found that 60.5% of women surveyed did not go outside during the first month after childbirth, while 30.4% of the women only went outside once or twice [23].

Due to the physical constraints of the “doing the month” practice, understandably, women who follow the custom closely often have to rely on help from family members or formal caregivers [24]. Though “doing the month” can cause significant discomfort, with some customs not supported by scientific evidence, a considerable number of young Chinese mothers still practice the ritual, following the customs of their ancestors [25]. It is common to receive support from husbands and senior members of a family during pregnancy and throughout the “doing the month” ritual [24], which can result in many family members living in the same household for some time. Although this arrangement can offer women substantial help, the extended time spent in close proximity may introduce a series of risk factors into a household [26]. Despite public smoking rates declining due to recent antismoking public policies, one unintended consequence is that smokers are more likely to smoke indoors [27], which affects people who may not be able to leave such an environment.

Deeply rooted in traditional Chinese culture is the consensus that young adults are expected to avoid correcting the behavior of seniors, even if the behavior is known to be health-damaging [28]. This cultural norm might be more pronounced when it comes to behaviors related to in-laws, so as to not appear confrontational and disrespectful. Regardless, these health-damaging behaviors, such as secondhand smoking, may harm women and their infants [28]. Furthermore, though women’s rights are steadily improving in China, it is essential to acknowledge that women’s overall welfare and well-being is still primarily overshadowed by that of men [29].

However, positive changes are occurring. A growing body of literature suggests that there has been a change in Chinese people’s attitudes and behaviors toward complying with traditional Chinese cultural values and social norms in recent years. Research finds that though the influence of traditional Chinese culture on Chinese social norms and practices (such as collectivism) is still ongoing and tangible, its hold over young adults is waning [30]. Furthermore, as the number of working women increases, more women gain financial freedom, bringing equal rights and gender equality to the forefront [31]. Overall, accumulating evidence indicates that values that are cherished by older Chinese generations might no longer be valued to the same degree by their younger counterparts [32].

## Measuring Smoking Around Expectant Mothers

This cultural shift may have an impact on how pregnant women address issues such as being exposed to harm through secondhand smoke from their husbands and older family members [33]. Due to recent cultural shifts, pregnant Chinese women are more likely to be aware of the devastating effects secondhand smoke can have on themselves and their unborn babies. These shifts may eventually result in mothers persuading their husbands or senior family members to change their smoking behaviors; mothers may even find a way to avoid these toxic environments filled with secondhand smoke. However, while this social phenomenon may be occurring, it is difficult to capture in a nonintrusive research setting [34]. Owing partially to ingrained cultural values and social norms, pregnant Chinese women may be reluctant to share their norm-defying behaviors toward their senior family members with researchers.

What might be possible, however, is to gauge this phenomenon from a different yet closely related angle. To this end, we propose a new method to gauge pregnant women’s rates of secondhand smoking. Different from traditional methods, which ask people how often they are exposed to secondhand smoke directly, we believe that a pregnant woman’s exposure to secondhand smoke may be more accurately gauged by asking about the smoking frequency of the woman’s family members (ie, husbands and other relatives) when they are in close proximity to the woman, especially during the “doing the month” period. In other words, there might be discrepancies in secondhand smoking rates reported by pregnant women due to deep-rooted cultural influences (eg, not wanting to accuse their family members, who play a pivotal role in the “doing the month” ritual, of reckless health behaviors that might harm the health and well-being of the expectant mothers, unborn children, and smokers themselves).

One way to gauge potential discrepancies in secondhand smoking rates reported by expectant mothers is by comparing these rates with smoking rates reported by the family members of these women. That is to say, rather than asking pregnant women about their exposure to secondhand smoke, more accurate information may be gleaned by directly asking family members about smoking frequency and duration in the presence of the pregnant woman. To further ensure that secondhand smoking faced by expectant mothers can be captured accurately, we believe it is important to collect data from expectant mothers and their family members separately (eg, survey conducted individually, rather than as a family unit), so that the role of social pressure in influencing survey results will be limited.

Protecting pregnant women from the harm of secondhand smoking safeguards the health and well-being of unborn children. Research that focuses on understanding women’s exposure to secondhand smoke and the various factors that contribute to their experience of secondhand smoking is urgently needed. The proposed approach is tangible and realistic from a research perspective. Understanding the relationship between traditional Chinese cultural values and social norms, women’s awareness of secondhand smoking and their background information (eg, education levels), and the women’s actual exposure to secondhand smoke from their husbands and other family members can help researchers obtain valuable insights needed to develop intervention measures to protect this vulnerable population [35].

## Concluding Remarks

In 2018, it is estimated that 4,285,033 new cancer cases were diagnosed in China, among which 1,919,023 were female [3]. Mounting evidence suggests that tobacco smoking increases the risk of many types of cancers [1]. Different from environmental factors such as air pollution, which might be more difficult to control and contain [36], tobacco smoking can be curbed in a timely and cost-effective manner [37], as seen in successful tobacco control interventions established in countries such as the United States. This insight is particularly promising for expectant mothers, as the adverse impacts of secondhand smoke are more pronounced for this population, and can harm both the mother’s and the infant’s health and well-being [13-18]. However, to develop a tailored and targeted tobacco control plan, health experts and government officials need to understand how much secondhand smoke expectant mothers are exposed to. We hope that the current proposed methods and future improved measures will lead to a better understanding of how much secondhand smoke expectant mothers are exposed to, as that knowledge is essential for designing and deploying effective interventions to protect expectant mothers and their infants from the harms of smoking and risk of cancer.
